# Identification and characterization of a novel *SCYL3-NTRK1* rearrangement in a colorectal cancer patient

**DOI:** 10.18632/oncotarget.19512

**Published:** 2017-07-24

**Authors:** Massimo Milione, Elena Ardini, Jason Christiansen, Emanuele Valtorta, Silvio Veronese, Roberta Bosotti, Alessio Pellegrinelli, Adele Testi, Filippo Pietrantonio, Giovanni Fucà, Ge Wei, Danielle Murphy, Salvatore Siena, Antonella Isacchi, Filippo De Braud

**Affiliations:** ^1^ 1st Division of Pathology, Department of Pathology and Laboratory Medicine, Fondazione IRCCS Istituto Nazionale dei Tumori, Milan, Italy; ^2^ Nerviano Medical Sciences S.r.l., Milan, Italy; ^3^ Ignyta, Inc., San Diego, CA, United States of America; ^4^ Division of Pathology, Department of Laboratory Medicine, Niguarda Cancer Center, Grande Ospedale Metropolitano Niguarda, Milan, Italy; ^5^ Dipartimento di Oncologia e Emato-Oncologia, Università degli Studi di Milano, Milan, Italy; ^6^ Medical Oncology Department, Fondazione IRCCS Istituto Nazionale dei Tumori, Milan, Italy

**Keywords:** NTRK1, TRKA, entrectinib, CRC, rearrangement

## Abstract

In colorectal cancer patients, chromosomal rearrangements involving *NTRK1* gene (encoding the TRKA protein) are shown in a small subset of patients and are associated with the constitutive activation of the kinase domain of TRKA. In turn, activated TRKA-fusion proteins are associated with proliferation and survival in colorectal cancer tumors.

Here we report the identification and functional characterization of a new *SCYL3-NTRK1* fusion gene in a 61-year-old colorectal cancer patient. To our knowledge, this fusion protein has never been previously documented in oncological patients. We show that this novel fusion is oncogenic and sensitive to TRKA inhibitors.

As suggested by other pieces of evidence, entrectinib - an orally available pan-TRK, ROS1 and ALK inhibitor - may have particular efficacy in patients with *NTRK* rearrangements. Therefore, screening for rearrangements involving *NTRK* genes may help identifying a subset of patients able to derive benefit from treatment with entrectinib or other targeted inhibitors.

## INTRODUCTION

Colorectal cancer (CRC) is the third most frequently diagnosed cancer worldwide, with more than one million new cases per year, and represents a major cause of cancer-related death [1]. Patients with advanced colon cancer are primarily treated with fluoropyrimidine-based systemic chemotherapy with or without irinotecan or oxaliplatin. Despite intense efforts dedicated to the identification of new and more effective therapies, many patients still have a poor treatment outcome. Although targeted agents such as cetuximab, panitumumab, bevacizumab, aflibercept, ramucirumab and regorafenib, introduced into the clinical practice during the last decade, have conferred incremental survival gains [2, 3] the identification of novel tumor targets and new targeted therapies is warranted to ensure a better long-term clinical benefit.

We originally reported the identification of a chromosomal rearrangement involving *NTRK1* (encoding the TRKA protein) and the *TPM3* gene in the KM12 colorectal tumor cell line and showed for the first time that the resulting TPM3-TRKA fusion protein is an oncogenic driver sensitive to TRKA inhibitors. We also showed by screening colorectal tumor samples that this rearrangement is present with approximately 1% frequency in CRC and that IHC with anti TRKA antibodies can be successfully applied to facilitate the initial screening of rearranged tumors [4]. These findings were further strengthened through genetic screenings performed on large collections of CRC specimens, confirming the presence of *NTRK1* rearrangements in 0.5–2% of CRC patients [5, 6]. Although many different *NTRK1* rearrangements have been already identified in a wide range of solid tumors (see www.ntrkfusions.com), only a limited number of fusion partners has been detected so far in CRC (*TPM3-NTRK1*, *LMNA-NTRK1* and *TPR-NTRK1* fusion genes) [5, 7, 8]. All these rearrangements result in the expression of fusion proteins harboring a constitutively activated TRKA kinase domain as a consequence of protein dimerization due to the presence of a coiled-coil domain in the N-terminal sequence of the partner protein. Preclinical data demonstrated that activated TRKA-fusion proteins are associated with/responsible for proliferation and survival in these subsets of CRC tumors [4, 6]. Importantly, we reported the first evidence of clinical benefit achieved with the TRKA-targeted agent entrectinib in a CRC patient bearing a *LMNA-NTRK1* positive tumor, providing clinical validation of activated TRKA as a target in CRC [7].

Based on these promising preclinical data and phase I results, TRKA-selected CRC patients are currently being enrolled in the open-label, multicenter global phase II basket study of entrectinib, an orally available pan-TRK, ROS1 and ALK inhibitor.

The molecular screening, aimed to select TRKA-positive patients for the enrollment in the above-mentioned trial, led to the discovery of a CRC patient harboring a novel *NTRK1* gene rearrangement consisting of a fusion between the *NTRK1* and *SCYL3* genes. Here we report the identification and characterization of the resulting *SCYL3-NTRK1* fusion gene that, to our knowledge, has not been previously reported in cancer patients.

## RESULTS

**Figure 1:**
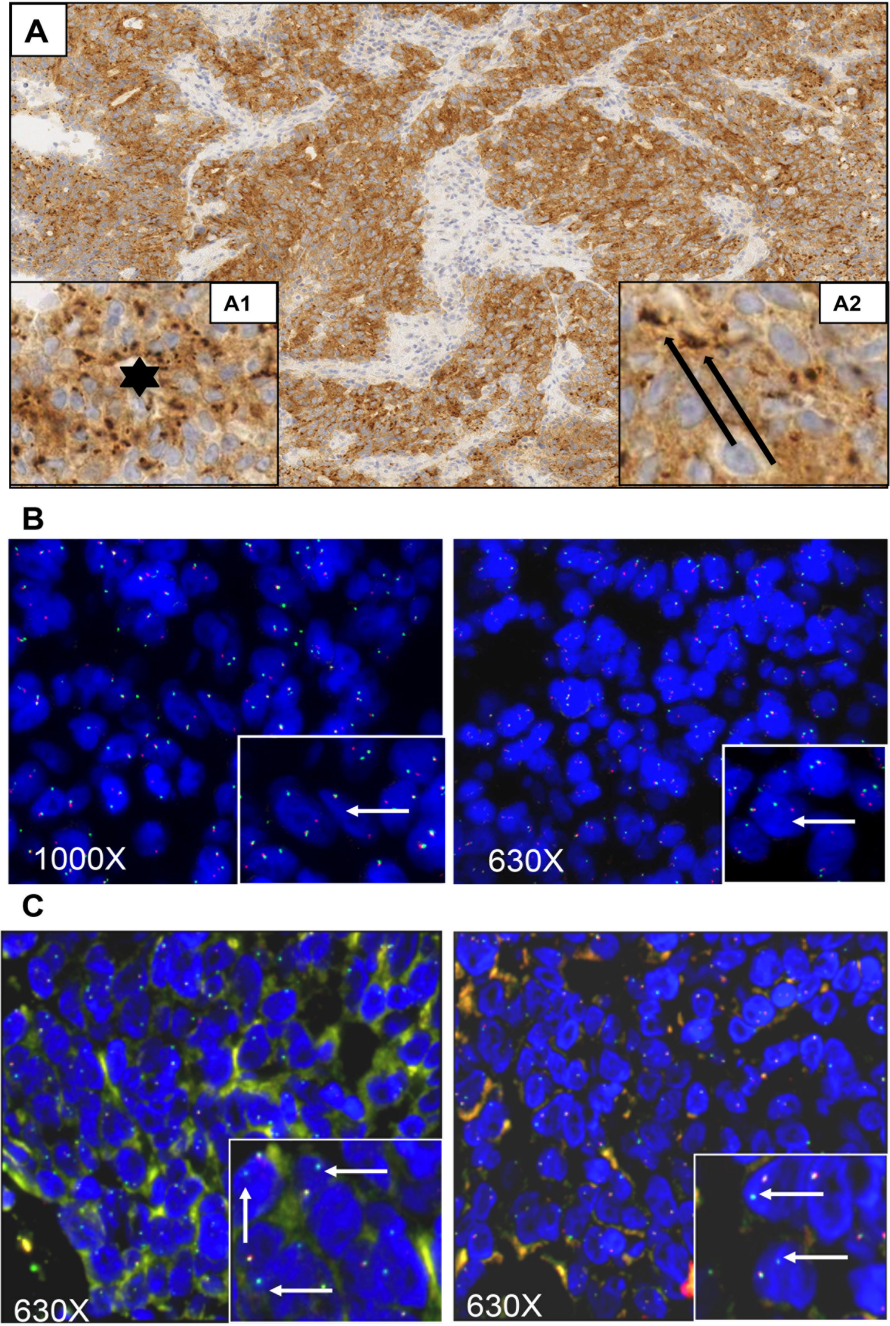
Histologic, immunohistochemical, and fluorescence-in-situ hybridization analyses of the case presented Immunohistochemical and Fluorescent-In-Situ Hybridization (FISH) images of poorly differentiated CRC. In immunohistochemical assays Panel (**A**), magnification 100X, whole neoplastic cells are stained by TRKA. The observed staining is characterized by a basic faint uniformly cytoplasmic staining associated to more intense staining organized in irregular round or ovoidal dark bodies (Insert A1, star: magnification 400X) preferentially distributed around nucleus. Dark bodies’ size is variable ranging from tiny dot spherules-like to bigger bodies with irregular size and shape. The bigger bodies are fused in coarse ovoid structure surrounding nucleus (Insert A2, arrow; magnification 400X). In panel (**B**), FISH analysis using the Abnova Break Apart probes showed the presence of one fusion signal along with separate green and orange signals suggesting the presence of a rearrangement of *NTRK1* gene. In Panel (**C**), FISH analysis performed with customized break-apart probes from Empire Genomics showed the green signal only, corresponding to the telomeric part of the break apart probe (white arrows inset), while the red signal corresponding to the centromeric probe (which covers the *NTRK1* gene) is missing. Magnification of images: 1000X or 630X as indicated.

The patient was a 61-year-old female diagnosed in February 2015 with adenocarcinoma of the right colon, infiltrating the pancreas. The patient progressed early on two standard treatment lines (FOLFOX-panitumumab followed by FOLFIRI-aflibercept). As part of a wide phase I screening program at our institutions (Fondazione IRCCS Istituto Nazionale dei Tumori and Niguarda Cancer Center, Milan, Italy), a deeper molecular characterization of the patient's primary tumor was performed. Results indicated that the tumor was wild type for RAS, BRAF, and EGFR with a high microsatellite instability (MSI-H) profile. At the time of progression to second line therapy (October 2015), the patient underwent endoscopic biopsy of the right-sided tumor mass as part of the pre-screening procedures for the enrollment in the phase I clinical trial ALKA-372-001 (EudraCT Number: 2012.000148-88) [9]. The tumor was tested by immunohistochemistry (IHC) for TRKA, ROS1 and ALK proteins whose expression may indicate the result of a genetic alteration. The IHC analysis revealed strong positivity for TRKA protein with a clear cytoplasmic distribution suggesting a potential aberrancy of *NTRK1* gene. The observed immunoreactivity was uniformly characterized by a basic faint cytoplasmic staining associated with a more intense staining organized in irregular or ovoidal clods, preferentially localized around the nuclei (Figure 1A). The size of the clods was highly variable and irregular in shape. Intriguingly, this immunoreactivity pattern appeared very different from the one observed in the patient harboring *LMNA-NTRK1* rearrangement, which was a moderate cytoplasmic staining associated with a more intense perinuclear staining as well as from the pattern found in *TPM3-NTRK1* bearing KM12 cells, where a diffused cytoplasmic staining could be appreciated (Supplementary Figure 2) [4, 7].

In order to verify if the detected TRKA expression was indeed the result of a genomic rearrangement, fluorescence *in situ* hybridization (FISH) analysis was performed using a commercial break-apart probe for the *NTRK1* gene (Abnova). Results from this analysis showed the presence of break-apart signal, with separate green and orange signals in 90% of analyzed nuclei, confirming the presence of a *NTRK1* rearrangement (Figure 1B). The FISH pattern was similar to the one previously detected in the KM12 cell line.

**Figure 2:**
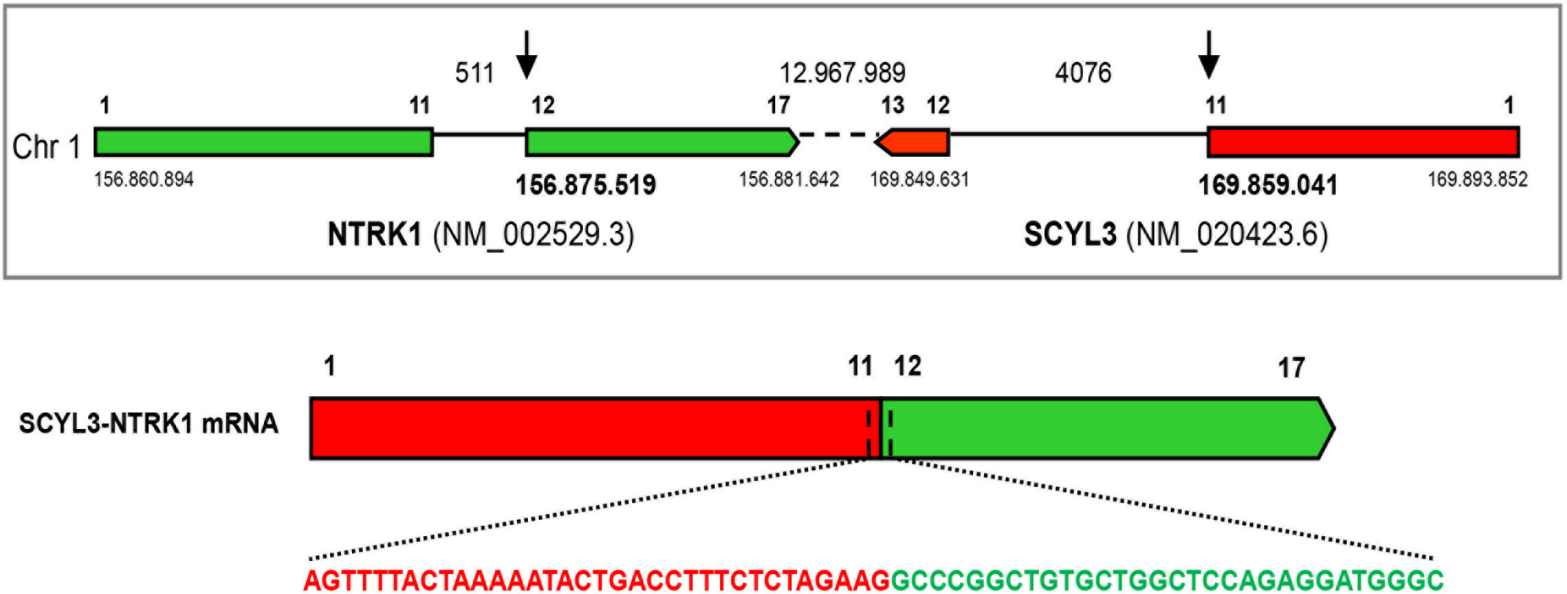
Identification of the SCYL3-NTRK1 gene rearrangement Schematic representation of the *SCYL3-NTRK1* rearrangement. Exons involved in the rearrangement are represented by colored boxes: *SCYL3* is reported in green, while *NTRK1* is reported in red. The sequence spanning the junction site is shown in detail.

Since the immunohistochemical staining of our sample was strikingly different from the one previously observed in the KM12 cells, we performed a second FISH analysis with customized break-apart probes (Empire Genomics), initially used for the identification of *LMNA-NTRK1* gene fusion in another CRC sample [7]. This further FISH analysis showed that the red centromeric signal from the break-apart probe was absent in 68% of analyzed nuclei, confirming once again the presence of a genetic alteration involving the *NTRK1* gene (Figure 1C). The observed discrepancy between the two hybridization patterns may be due to the specific features of the probes used in the two FISH analyses. In particular the loss of the red-labeled centromeric probe observed in the second analysis only can be explained with the different length of the centromeric probe. Indeed, differences in FISH probe design have previously been shown to complicate the evaluation of the small interstitial deletion that forms the *GOPC-ROS1* fusion [10]. Taken together, these observations suggested the presence of a novel *NTRK* rearrangement and prompted further molecular characterization for the identification of the N-terminal partner gene. An RNA-based anchored multiplex polymerase chain reaction Next Generation Sequencing (NGS) assay, customized for the detection of rearrangements of selected tyrosine kinases and for simultaneous identification of the fusion partner, was applied [11]. This approach allowed the identification of a new *NTRK1* rearrangement resulting from an inversion within chromosome 1 fusing exons 1–11 of the *SCY1 Like Pseudokinase 3* (*SCYL3*) gene with exons 12–17 of *NTRK1* gene (Figure 2). No other *NTRK1* fusion transcripts were observed.

**Figure 3:**
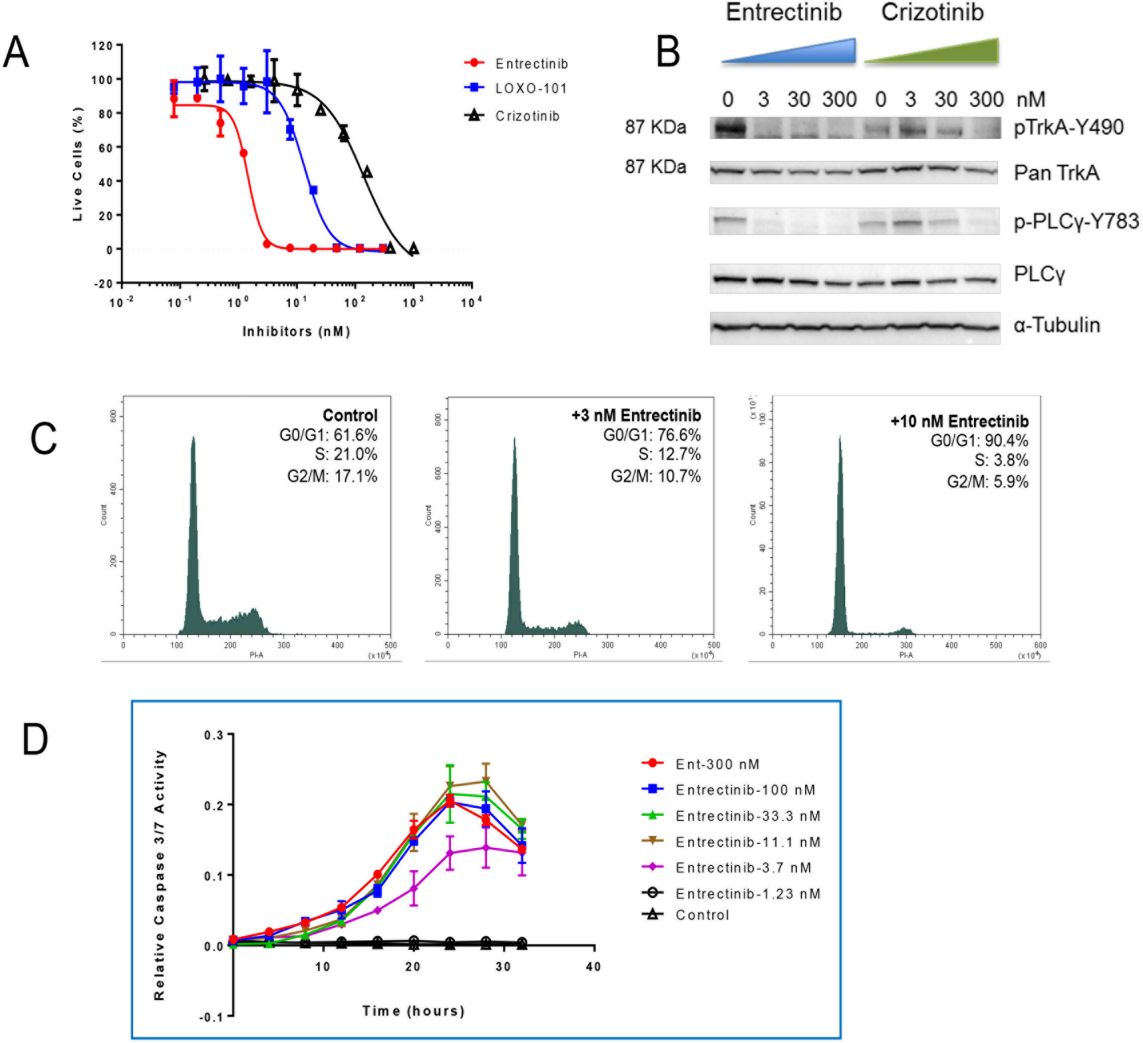
Mechanism of action of entrectinib Confirmation that the *SCYL3-NTRK1* fusion gene can be targeted by TRK inhibitors. (**A**) Comparison of anti-proliferation activities of 3 tyrosine kinase inhibitors in Ba/F3-SCYL3-NTRK1 cell line. (**B**) Western blot analysis of the changes in phosphorylation levels of TRKA and its downstream transducer PLCγ 2 hours post entrectinib and crizotinib treatment in Ba/F3-SCYL3-NTRK1 cells. (**C**) Cell cycle analysis of Ba/F3-SCYL3-NTRK1 treated with 3 and 10 nM of entrectinib for 18 hours compared to untreated cells. (**D**) Relative caspase 3/7 activities in Ba/F3-SCYL3-NTRK1 cells treated with increasing concentrations of entrectinib over time.

As this rearrangement has never been previously described, to formally demonstrate its oncogenic potential, we transfected IL3-dependent Ba/F3 cells (a murine pro-B system that does not possess any endogenous human TRKA expression) with *SCYL3-NTRK1* cDNA construct (Supplementary Figure 1). As consequence of the expression of the corresponding TRKA-containing fusion protein, Ba/F3 cells acquired IL3-independent proliferation capability, demonstrating that *SCYL3-NTRK1* is indeed an oncogenic driver. These transformed cells were used to test and compare the potency of TRK inhibitors [6, 9]. Treatment with three different TRK inhibitors strongly affected the proliferation of Ba/F3-SCYL3-NTRK1 whereas no growth inhibition was observed in the parental Ba/F3 cells or the Ba/F3-SCYL3-NTRK1 cells grown in the presence of IL-3 [12]. Entrectinib was found to be the most potent compound inhibiting the proliferation of TRKA-driven cells with an IC50 value of 1.5 nM. Other TRK inhibitors LOXO-101 and Crizotinib were also able to decrease the proliferation of Ba/F3-SCYL3-NTRK1 cells with IC_50_ values of 11.2 nM and 160 nM, respectively (Figure 3A). The mechanism of action of entrectinib was confirmed by flow cytometer analysis of Propidium Iodide (PI) stained cells 18-hour post entrectinib treatment. Consistent with previous publication [13], entrectinib induced cell cycle arrest at G0/G1 (Figure 3C). Furthermore, caspase 3/7 activities were peaked between 24 to 30 hours upon entrectinib treatment, at as low as 3.7 nM (Figure 3D).

Western Blot analysis demonstrated a dose-dependent modulation of TRKA phosphorylation with concomitant inhibition of phosphorylation of the downstream signal transducer PLCγ (Figure 3B), consistent with prior studies [13]. Crizotinib was also able to modulate TRKA signaling, although at significantly higher doses, in agreement with its calculated IC_50_.

## DISCUSSION

The identification of oncogenic fusions resulting from chromosomal rearrangements in many different tumor types represents an important opportunity for novel therapeutic strategies. This is definitely true for lung adenocarcinoma, as the introduction into the clinical practice of therapeutic approaches based on the specific targeting of activated kinases has dramatically changed the clinical outcome of many patients [14, 15]. By contrast, in a high un-met medical need such as CRC, the era of targeted therapy coupled with positive predictive biomarkers, is still in its infancy. For many years molecular analyses performed in metastatic CRC patients have been aimed mainly to identify *RAS* or *BRAF* mutations that are associated with the lack of response to anti-EGFR treatment [16, 17]. The identification of positive predictive biomarkers to be exploited for targeted therapeutic approaches in CRC, such as HER2 for dual HER2 blockade, MSI-H for immune checkpoint inhibitors, BRAF for dual/triple inhibition strategies and *MGMT* methylation for alkylating agents [18–21], represents a paradigm shift for the future treatment landscape [22]. The identification of chromosomal rearrangements involving *NTRK1* as a low frequency event in a subset of CRC patients is a relatively recent finding [23]. So far limited efforts have been dedicated to the screening of a high number of cases in order to define the prevalence and the relevance of these findings for CRC patient population. The clinical validation of *NTRK1* rearrangements as targets for therapy in CRC will spur the molecular screening of an increasing number of patients, widening the opportunity for the application of TRKA-targeted agents into the clinical practice.

In our laboratories, the presence of *NTRK1* rearrangements is routinely evaluated by IHC in all CRC tumors in the course screening for patients’ enrollment into clinical trials with entrectinib. This screening previously resulted in the identification of two oncogenic fusions, *TPM3-NTRK1* and *LMNA-NTRK1*, that were fully characterized. We also demonstrated that the patients harboring TRK rearrangements derive clinical benefit from the treatment with entrectinib, a specific TRK inhibitor [9]. Here we report the identification of a new chromosomal rearrangement involving *NTRK1* that results in the generation of the chimeric SCLY3-TRKA fusion protein. Despite the significant number of different fusion partners that has already been identified for *NTRK1* in solid tumors, to our knowledge this is the first time that the *SCYL3* gene is found to be involved in a rearrangement with *NTRK1* or any other kinase gene. It will be interesting to understand if this fusion is specific to CRC or it is also present in other tumor types, such as non small cell lung cancer, papillary thyroid carcinoma and glioblastomas, where multiple *NTRK1* rearrangements have already been identified [23–24].

*SCYL3*, also known as *PACE-1*, a gene widely expressed in a variety of tissues, encodes for a protein whose function is still poorly understood. The full-length protein is able to bind to ezrin through a C-terminal domain determining its localization at the level of lamellipodia, a specialized cell structure that facilitates cell advancement across a substrate, where it probably plays a role in cell spreading and motility. In addition the N-terminal region contains a myristoylation motif that is responsible for its association with the Golgi apparatus. Intriguingly, SCYL3 protein contains a kinase domain but any attempt to demonstrate its intrinsic kinase activity failed so far, suggesting that it should be probably considered a pseudokinase [26].

As the *SCYL3* gene maps to chromosome 1q23.3-4 (1q24.2 on NCBI, OMIM, Gene Cards) we hypothesize that the generation of the fusion gene *SCYL3-NTRK1* is a consequence of an intrachromosomal inversion that juxtaposes the first 11 exons of *SCYL3* to a portion encompassing exon-12-17 of *NTRK1*. As a consequence of this fusion event SCYL3 loses the C-terminal sequence responsible for ezrin binding but it maintains its myristoylation motif. The expected localization of the resulting fusion protein SCYL3-TRKA at the level of the Golgi apparatus is compatible with the characteristic punctate intracellular staining pattern observed by immunohistochemical analysis [27]. The *in vitro* data clearly demonstrate that the *SCYL3-NTRK1* fusion gene is oncogenic and that the resulting fusion protein has constitutive kinase activation. Targeted inhibitors, in particular entrectinib, strongly inhibit the SCYL3-TRKA phosphorylation leading to cell growth inhibition and confirming the role of the expressed fusion protein as driver for proliferation. Our data demonstrate that entrectinib represents an important potential opportunity for the treatment of patients whose tumors harbor this new *NTRK1* rearrangement. Unfortunately, the clinical conditions of this patient rapidly degraded because of tumor progression and it was not possible to treat her with entrectinib.

However, consistent with prior published reports of tumor growth inhibition in *NTRK1* rearranged patients treated with entrectinib [7–9], our data showed that this drug has a significant therapeutic potential against newly identified *NTRK1* gene rearrangements. Consequently, the screening of CRC for the detection of gene rearrangements involving the *NTRK1*, *NTRK2* and *NTRK3* genes has the ability to identify a limited subset of patients able to derive benefit from treatment with entrectinib or other targeted inhibitors.

## MATERIALS AND METHODS

### Immunohistochemistry

TRKA protein expression was assessed in formalin-fixed paraffin-embedded (FFPE) tumor tissue sections using a rabbit monoclonal antibody (clone EP1058Y; Epitomics, Burlingame, CA) that recognizes the carboxyl-terminus of the protein. Briefly, three micron-thick sections were reacted for 30 min with the anti-TRKA antibody used at 1:200 working dilution and then incubated with a commercially available detection kit (EnVision™ FLEX+, Dako, Glostrup, Denmark) following the manufacturer's instructions and previously refined IHC methods. The specificity of all reactions was checked replacing the primary antibody with a non-related mouse immunoglobulin at comparable dilutions or using normal serum alone, Positive and negative controls were used as appropriate KM12 cell line, known to carry a *TPM3-NTRK1* gene rearrangement, was used as positive control.

### FISH analysis for NTRK1

In order to identify possible rearrangements involving the *NTRK1* gene, dual colour FISH analysis was performed on 3 μm-thick sections of FFPE tumor tissue using a commercially available, specific break-apart probe kit (Abnova) and following the manufacturer's instructions. In addition customized break-apart probes, (Empire Genomics), previously described, were used [7]. Briefly, *NTRK1* FISH analysis was conducted using 10 μl mix-probe made up by 1 ul BAC (Bacterial Artificial Chromosome) genomic probe RP11-349I17 (1q23.1) (206 kb) labelled in SpectrumOrange (Empire Genomics), 1 ul BAC (Bacterial Artificial Chromosome) genomic probe RP11-1038N13 (1q23.1) (129 kb) labelled in SpectrumGreen (Empire Genomics), 1 ul of sterile water and 7 ul of hybridisation buffer (Empire Genomics). Probes and target DNA of specimen were co-denatured in HYBRite System (DakoGlostrup, Denmark) for 5 min at 75°C and then hybridized overnight at 37°C. Slides were washed with post-hybridisation buffer (DakoGlostrup, Denmark) (73°C for 2 min) and counterstained with 4,6-diamino-2phenylindole (DAPI II; Vysis, Vysis, Downers Grove, IL. USA). A sample was scored as positive if rearrangements of *NTRK1* gene were detected in at least 15 of 100 analysed nuclei. Healthy tissue was used as internal negative control. Fluorescent *in situ* hybridisation signals were evaluated with a Zeiss Axioscope Imager Z1 (Zeiss, Gottingen, Germany). Images for documentation were captured with CCD camera and processed using the MetaSystems Isis software.

### Targeted RNA sequencing

Gene rearrangements/fusions were analyzed by targeted RNA sequencing, using an anchored multiplex PCR (AMP) method (ArcherDx, Inc.) as previously described [11]. Briefly, a custom panel was generated to focus on determination of gene rearrangements in *NTRK1*, *NTRK2*, *NTRK3*, *ROS1*, and *ALK* and *RET* genes (encoding the TRKA, TRKB, TRKC, ROS1, ALK and RET proteins) along with associated housekeeping genes to assess RNA fragmentation. The AMP method allows for improved sensitivity to novel fusion partners due to an initial adapter ligation step that facilitates priming without de novo knowledge of the gene rearrangement partner and limitations from the small fragments generated in FFPE tissues.

This study was performed according to the clinical standards of the 1975 and 1983 Declaration of Helsinki and was approved by the Ethical Committee of Fondazione IRCCS Istituto Nazionale dei Tumori (n° INT 48/16).

### Functional studies of SCYL3-NTRK1 fusion gene

cDNAs (see supplementary data) encoding the SCYL3-NTRK1 fusion protein was inserted into the lentiviral vector pVL-EF1a-MCS-IRES-Puro (BioSettia, San Diego, CA) and introduced into the murine, IL-3 dependent, pro-B Ba/F3 cells (DSMZ) [12]. Ba/F3-SCYL3-NTRK1 stable cells only proliferate without the need of IL-3. Proliferation assays were conducted with these cells with various concentrations of entrectinib (synthesized by Ignyta), LOXO-101 (synthesized by Ignyta) and crizotinib (purchased from Sellekchem). Plates were incubated at 37°C in 5% CO_2_ for 72 hours, after which cell viability was assessed by measuring ATP content using Cell Titer-Glo^®^ Luminescent Cell Viability assay (Promega). IC_50_s were calculated using the 4-parameter variable slope curve fit (GraphPad Prism).

For Western blot analysis, cell lysates were prepared in 1X RIPA buffer (EMDMillipore) with protease and phosphatase inhibitors (EMDMillipore) 2 hours post treatment. The protein lysates were analyzed with SDS-PAGE gels (Thermofisher) and transferred to the PVDF membrane using iBlot2 (Thermofisher). The western blot analysis was performed using the primary antibodies (phosphorylated/total TRKA and PLCγ, and α-tubulin) from Cell Signaling Technology and the HRP-labeled secondary antibodies were purchased from LI-COR. The reactive bands were developed by ECL Prime reagent (Amersham) and images were captured by ChemiDoc (Bio-Rad).

To evaluate the effect of entrectinib on the cell cycle, cells were treated with various concentrations of entrectinib for 18 hours and fixed in cold 70% ethanol, and stained by Propidium Iodide (PI/RNase Staining Buffer (BD Bioscience, cat#550825)). The cell cycles were analyzed using a CytoFlex flow cytometer (Beckman Coulter).

To confirm the induction of apoptosis by entrectinib, cells were treated with various concentrations of entrectinib and Caspase-3/7 Reagent (IncuCyte, #4440) was added to all samples. Live images (Phase and green fluorescent channels) were collected at 4-hour intervals over 40 hours. Active Caspase-3/7 activities were normalized to cell numbers.

## SUPPLEMENTARY MATERIALS FIGURES


