# Supplementary material for: Identification and characterization of a novel SCYL3-NTRK1 rearrangement in a colorectal cancer patient
Source: Oncotarget. 2017 Jul 24;8(33):55353–60. doi: 10.18632/oncotarget.19512 (PMC5589663; doi:10.18632/oncotarget.19512)
Supplement: Supplementary file 1 [file oncotarget-08-55353-s001.pdf]

Identification and characterization of a novel *SCYL3-NTRK1* rearrangement in a colorectal cancer patient

SUPPLEMENTARY MATERIALS

>SCYL3:NTRK1:11:12:NM-020423:NM-002529

ATGGGATCAGAGAACAGTGCTTTAAAGAGCTATACACTGAGAGAACCACCATTTACCTTACC
CTCTGGACTTGCTGTTTATCCCGCTGTACTGCAAGATGGCAAATTTGCTTCAGTTTTTGTGTA
TAAGAGAGAAAATGAAGACAAGGTTAATAAAGCTGCCAAGCATTTGAAGACACTTCGTCAC
CCTTGCTTGCTAAGATTTTTATCTTGTACTGTGGAAGCGGATGGCATTCATCTTGTCACTGAG
CGAGTACAGCCCCTGGAAGTGGCTTTGGAAACATTGTCTTCTGCAGAGGTCTGTGCTGGGAT
CTATGACATATTGCTGGCTCTTATCTTCCTTCATGACAGAGGACACCTAACACACAATAATGT
CTGTTTATCATCTGTGTTTGTGAGTGAAGATGGACACTGGAAGCTAGGAGGAATGGAAACTG
TTTGTAAAGTTTCTCAGGCCACACCAGAGTTTCTGAGGAGTATTCAGTCAATAAGAGACCCA
GCATCTATCCCTCCTGAAGAGATGTCTCCAGAATTCACAACTCTCCCAGAGTGTCATGGACA
TGCCCGGGATGCCTTTTCATTTGGAACATTGGTGGAAAGTTTGCTCACAATCTTAAATGAAC
AGGTTTCAGCGGATGTTCTCTCCAGCTTTCAACAGACCTTGCACTCAACTTTGCTGAATCCCA
TTCCAAAATGTCGGCCAGCGCTCTGCACCTTACTATCTCATGACTTCTTCAGAAATGATTTTC
TGGAAGTTGTGAATTTCTTGAAAAGTTTAACATTGAAGAGTGAAGAGGAGAAAACGGAATT
CTTTAAATTTCTGCTGGACAGAGTCAGCTGCTTGTCAGAGGAATTGATAGCTTCAAGGTTGG
TGCCTCTTCTGCTTAATCAGTTGGTGTTTGCAGAGCCAGTGGCTGTTAAGAGTTTTCTTCCTT
ATCTGCTTGGCCCCAAAAAAGATCATGCGCAGGGAGAAACTCCTTGCTTGCTCTCACCAGCC
CTGTTCCAGTCACGGGTGATCCCCGTGCTTCTCCAGTTGTTTGAAGTTCATGAAGAGCATGTG
CGGATGGTGCTGCTGTCTCACATCGAGGCCTACGTGGAGCACTTCACTCAGGAGCAGCTGAA
GAAAGTCATCTTGCCACAGGTTTTGCTGGGCCTGCGTGATACTAGCGATTCCATTGTGGCAA
TTACTCTGCATAGCCTAGCAGTGCTGGTCTCTCTGCTTGGACCAGAGGTGGTTGTGGGAGGA
GAACGAACCAAGATCTTCAAACGCACTGCCCCAAGTTTTACTAAAAATACTGACCTTTCTCT
AGAAG|GCCCGGCTGTGCTGGCTCCAGAGGATGGGCTGGCCATGTCCCTGCATTTCATGACAT
TGGGTGGCAGCTCCCTGTCCCCCACCGAGGGCAAAGGCTCTGGGCTCCAAGGCCACATCATC
GAGAACCCACAATACTTCAGTGATGCCTGTGTTCACCACATCAAGCGCCGGGACATCGTGCT
CAAGTGGGAGCTGGGGGAGGGCGCCTTTGGGAAGGTCTTCCTTGCTGAGTGCCACAACCTCC
TGCCTGAGCAGGACAAGATGCTGGTGGCTGTCAAGGCACTGAAGGAGGCGTCCGAGAGTGC
TCGGCAGGACTTCCAGCGTGAGGCTGAGCTGCTCACCATGCTGCAGCACCAGCACATCGTGC
GCTTCTTCGGCGTCTGCACCGAGGGCCGCCCCCTGCTCATGGTCTTTGAGTATATGCGGCAC
GGGGACCTCAACCGCTTCCTCCGATCCCATGGACCTGATGCCAAGCTGCTGGCTGGTGGGGA
GGATGTGGCTCCAGGCCCCCTGGGTCTGGGGCAGCTGCTGGCCGTGGCTAGCCAGGTCGCTG
CGGGGATGGTGTACCTGGCGGGTCTGCATTTTGTGCACCGGGACCTGGCCACACGCAACTGT
CTAGTGGGCCAGGGACTGGTGGTCAAGATTGGTGATTTTGGCATGAGCAGGGATATCTACAG
CACCGACTATTACCGTGTGGGAGGCCGCACCATGCTGCCCATTCGCTGGATGCCGCCCGAGA
GCATCCTGTACCGTAAGTTCACCACCGAGAGCGACGTGTGGAGCTTCGGCGTGGTGCTCTGG
GAGATCTTCACCTACGGCAAGCAGCCCTGGTACCAGCTCTCCAACACGGAGGCAATCGACTG
CATCACGCAGGGACGTGAGTTGGAGCGGCCACGTGCCTGCCCACCAGAGGTCTACGCCATC
ATGCGGGGCTGCTGGCAGCGGGAGCCCCAGCAACGCCACAGCATCAAGGATGTGCACGCCC
GGCTGCAAGCCCTGGCCCAGGCACCTCCTGTCTACCTGGATGTCCTGGGCTAG

Supplementary Figure 1: The cDNA sequence of *SCYL3-NTRK1*.

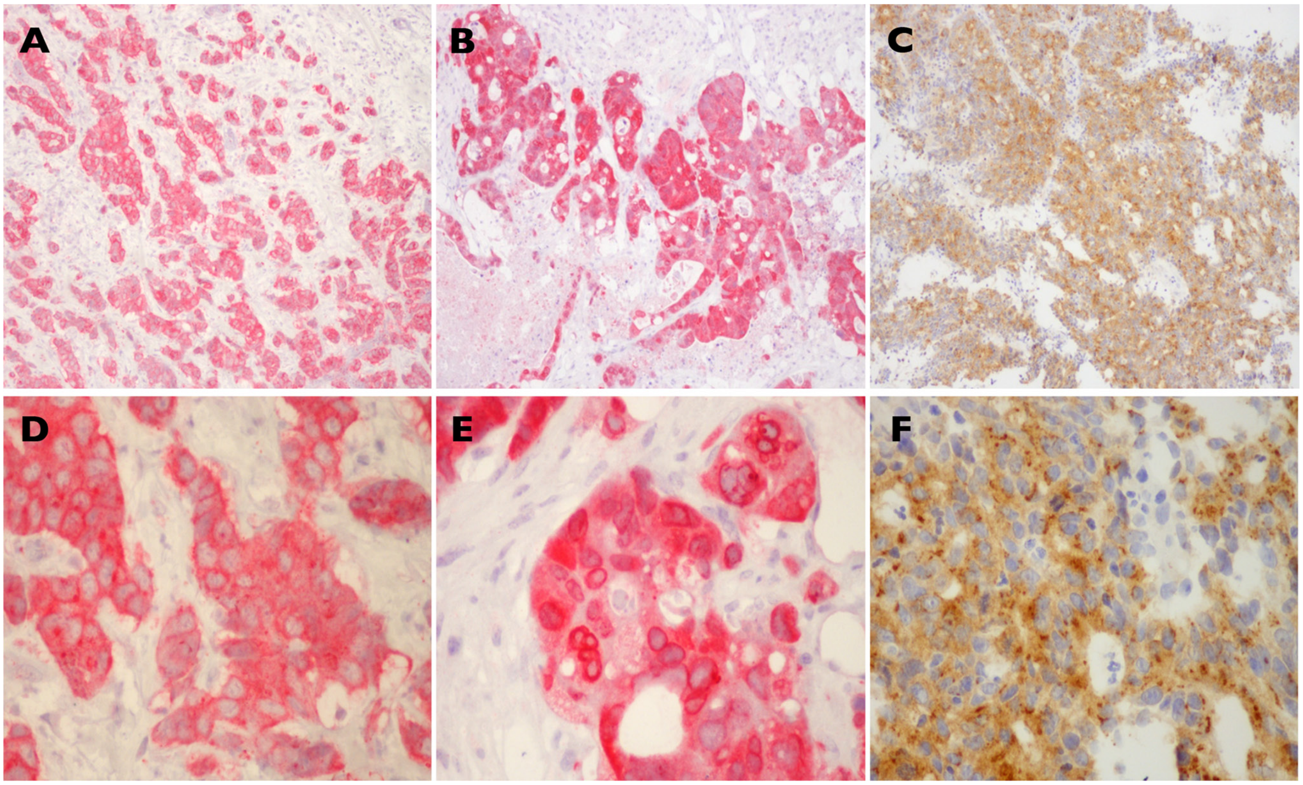

Supplementary Figure 2: TRKA staining in relationship to gene rearrangement. TRKA pattern staining identified in relationship to different *NTRK1* rearrangements (**A** and **D**). Tumor cells harboring the *TPM3-NTRK1* gene rearrangement show a strong cytoplasmic staining (**B** and **E**). Tumor cells harboring the *LMNA-NTRK1* gene rearrangement show a cytoplasmic staining associated with a more intense staining localized at nuclear membrane (**C** and **F**). Tumor cells harboring the *SCYL3-NTRK1* gene rearrangement show a moderate cytoplasmic staining associated with a more intense staining organized in irregular or ovoidal clods localized around nuclei; A, B, C 100X magnification; D, E, F 400X magnification. A, B, D, E: detection with chromogenic substrate FAST RED (red), (C and F) detection with chromogenic substrate DAB (brown).
